# Using Heart Rate Variability Methods for Health-Related Outcomes in Outdoor Contexts: A Scoping Review of Empirical Studies

**DOI:** 10.3390/ijerph20021330

**Published:** 2023-01-11

**Authors:** Jonah D’Angelo, Stephen D. Ritchie, Bruce Oddson, Dominique D. Gagnon, Tomasz Mrozewski, Jim Little, Sebastien Nault

**Affiliations:** 1 School of Kinesiology and Health Sciences, Laurentian University, Sudbury, ON P3E 2C6, Canada; 2 Center for Research in Occupational Safety and Health, Sudbury, ON P3E 2C6, Canada; 3 Center for Rural and Northern Health Research, Sudbury, ON P3E 2C6, Canada; 4 Laurentian Research Institute for Aging, Laurentian University, Sudbury, ON P3E 2C6, Canada; 5 Faculty of Sport and Health Sciences, University of Jyväskylä, 40014 Jyväskylä, Finland; 6 Digital Scholarship Infrastructure Department, York University, Toronto, ON M3J 1P3, Canada

**Keywords:** Heart Rate Variability (HRV), RR Interval, Autonomic Nervous System (ANS), outdoors, nature, wilderness, health, well-being, wellness, scoping review

## Abstract

Heart rate variability (HRV) is a psychophysiological variable that is often used in applied analysis techniques to indicate health status because it provides a window into the intrinsic regulation of the autonomic nervous system. However, HRV data analysis methods are varied and complex, which has led to different approaches to data collection, analysis, and interpretation of results. Our scoping review aimed to explore the diverse use of HRV methods in studies designed to assess health outcomes in outdoor free-living contexts. Four database indexes were searched, which resulted in the identification of 17,505 candidate studies. There were 34 studies and eight systematic reviews that met the inclusion criteria. Just over half of the papers referenced the 1996 task force paper that outlined the standards of measurement and physiological interpretation of HRV data, with even fewer adhering to recommended HRV recording and analysis procedures. Most authors reported an increase in parasympathetic (n = 23) and a decrease in systematic nervous system activity (n = 20). Few studies mentioned methods-related limitations and challenges, despite a wide diversity of recording devices and analysis software used. We conclude our review with five recommendations for future research using HRV methods in outdoor and health-related contexts.

## 1. Introduction

The outdoors is a place commonly utilized by adventure seekers and explorers; however, recent evidence suggests there is a relationship between contact with nature and an improvement in health and well-being [1,2,3,4,5,6,7]. These health benefits have been observed through the analysis of many physiological and psychological outcomes, primarily relating to stress and relaxation [1,8]. Stress and relaxation can be monitored by analyzing cardiovascular activity via heart beat-to-beat variations, known as Heart Rate Variability [8,9,10,11,12]. Heart Rate Variability (HRV) is a psychophysiological measure that is often used in studies to monitor the activation of the autonomic nervous system (ANS). HRV analysis techniques can differentiate between activation of the parasympathetic nervous system (PSNS), suggesting a relaxed state that reflects good health and the sympathetic nervous system (SNS), suggesting a stressed state that leads to ill health when chronically activated [11]. Heart rate is the average beats-per-minute (BPM) that the heart beats, whereas HRV reflects the variability of heart rate over time [11]. Through an array of techniques, HRV can be analyzed to produce derived variables that reflect health status [9,10,11] (see Table 1 below). Additionally, HRV can be collected in a free-living or field setting using a small portable (or wearable) device [13]. This presented the opportunity for HRV methods to be used to determine health-related outcomes in diverse outdoor settings. However, as HRV methods started to become more widely used nearly three decades ago, there were many questions surrounding the standards of measurement, physiological interpretation, and guidelines for clinical use [11].

In 1996, a joint task force of the European Society of Cardiology and the North American Society of Pacing and Electrophysiology published standards and recommendations for measurement, analysis, and interpretation of HRV data [11]. The first recommendation was that equipment used to collect electrocardiogram (ECG) data should adhere to standards such as signal, noise ratio, common mode rejection, and bandwidth [11]. Second, studies should use 5-min recordings for frequency domain analysis and 24-h recordings for time domain methods [11]. Third, visual inspection and manual corrections should be made to the R-R intervals from the ECG data, when using both time domain and frequency domain analysis [11]. Task force authors concluded that HRV has the potential to reflect the role of the autonomic nervous system in patients who are healthy or in those who have cardiovascular disorders [11]. Thus, the two most common HRV analysis techniques are time domain and frequency domain. Time domain analysis focuses on the difference in R-R intervals over time [11]. Frequency domain analysis uses assigned frequency bands, and the number of normal-to-normal intervals within each band is then counted and compared to the number in other bands [11]. The main variables associated with these two analysis techniques are presented in Table 1 [11].

Following the task force recommendations, evidence began to emerge after 1996 showing the health benefits of immersion in outdoor contexts using HRV as a psychophysiological variable. Studies related to Shinrin-yoku (forest bathing) [14] and green exercise [1] used HRV methods. Shinrin-yoku involves participants taking in the forest atmosphere, and using their senses, to improve their mental and physical well-being [14]. A review of forest bathing studies revealed that Shinrin-yoku has many therapeutic benefits, which are reflected in positive outcomes related to the immune system, cardiovascular system, respiratory system, and mental wellness [1,14,15]. These effects are believed to come from interaction within the forest greenspaces and the natural stimuli associated with these environments [16]. Green exercise focuses on the known benefits of physical activity and combines it with the benefits of the natural environment. Essentially, green exercise involves physical activity in a natural outdoor setting [17].

Clinically, HRV analysis is particularly helpful in understanding cardiovascular health [11,12,18,19]; stress is a variable that significantly impacts cardiovascular response [20], and this can be assessed using HRV methods [21]. Decreased HRV is a risk factor for mortality, and higher HRV is associated with reduced risk for several chronic diseases, such as coronary artery disease [21]. Thus, HRV can help predict the health of the cardiovascular system as mediated through the ANS [18]. The health outcomes related to participation in outdoor environments have become an increasing area of interest over the last decade [22], primarily as they relate to stress reduction and increased relaxation. This has led to HRV methods being used in studies designed to understand health outcomes related to immersion in outdoor environments involving free-living conditions [19].

When HRV data are collected in an outdoor field setting, the data can be analyzed and interpreted in different ways based on the methodological approach used (see Table 1). However, there is little guidance related to the best methods for analyzing HRV in the various outdoor free-living contexts. Thus, this scoping review of empirical research aimed to identify and assess the various HRV methods used in an outdoor and/or natural environment that were designed to relate to a health outcome. This study was designed to address three research questions (RQs): (1) What are the characteristics of studies using HRV methods in outdoor and health-related contexts? (2) What types of HRV analysis and outcome measures are used in these studies? and (3) What are the methodological challenges and study limitations reported in these studies?

## 2. Methods

A scoping review method was selected to address our purpose and research questions based on the diversity and complexity of HRV data collection techniques for health-related outcomes in outdoor contexts. According to Munn et al., scoping reviews are “useful tools to investigate the design and conduct of research on a particular topic” [23]. They are also valuable for emerging fields or when there is diversity and complexity [24,25]. Arksey et al. were the first to publish methodological guidance for scoping reviews [26], and this was updated in 2010 [27] and again in 2018 [23].

### 2.1. Literature Search

Two searches were conducted: the first occurred on 27 October 2018, and the second on 15 May 2022. The second search followed the same protocols and used the exact search string as the first search, and it was completed to update the study before submission for publication. The following indexes were searched: PubMed, Web of Science, EBSCO Platform, and CINAHL Platform. No limits were set during the initial search. However, papers prior to 1996 and those published in any language other than English were removed by the reviewer(s) upon the initial screening. Initial search terms were selected based on a review of relevant/associated papers and were compared against relevant MESH terms. The final derived search string used to obtain studies was created iteratively to ensure that only relevant terms were used, and other keywords excluded (e.g., NOT…) to ensure irrelevant studies were not included. The final search string used in the initial PubMed index search appears in (Appendix A); it was then translated to search the additional three indexes (Web of Science, CINAHL, and EBSCO).

### 2.2. Screening and Selection Criteria

The initial eligibility assessment focused on screening titles and abstracts and removing duplicates; a single researcher completed it. Following full-text extraction of the remaining studies, they were assessed by the researcher for inclusion and then screened by a second author until consensus was achieved on the included studies. A final citation chaining process identified relevant papers from the reference lists of the included systematic reviews. The screening and selection process used five inclusion criteria:

1. Date range: Studies published from 1996 to 15 May 2022 (date of our last search) to reflect the year when HRV standards were first published (1996) [11];
2. Empirical studies: Systematic reviews and original studies involving primary data collection related to the three variables (outdoor environment, HRV methods, health outcomes);
3. Outdoor environment: The setting was in a wilderness, outdoor, or natural environment; contact with nature was the primary context for the intervention or program. The immersive experience had no minimum or maximum limit of duration;
4. HRV methods: Methods involved HRV devices, data collection, and analysis techniques;
5. Health outcomes: Primary outcomes were associated with therapeutic or holistic health-related benefits; these outcomes were derived from HRV data and analysis.

### 2.3. Data Extraction and Charting

A single researcher performed the initial data extraction and charting. The following extraction fields were obtained from each paper: title, author(s), date of publication, type of study design, population size, outdoor context, intervention, control/comparison, HRV data collection methods and analysis, setting, challenges, limitations, ethical considerations, and risk of bias. Extracted data were charted in a Microsoft Excel spreadsheet to provide a visual map of the extracted fields for all the included studies.

### 2.4. Data Analysis

To address RQ1, data analysis involved using summary counts and percentages for method characteristics from extraction fields containing quantitative data (e.g., date of publication) or data that could be quantified (e.g., type of study). To address RQ2, data were analyzed and summarized quantitatively (counts and percentages) for the characteristics of HRV analysis procedures and outcome variables in terms of time domain or frequency domain analysis (See Table 1). Outcome data were also assessed in terms of author claims of the impact on SNS, PSNS, and health. To address RQ3, a thematic analysis [25] was used to summarize the challenges and limitations identified by the authors of each study. Systematic reviews were analyzed separately. The summary data tables addressing each RQ and findings from the systematic reviews were discussed and reviewed in a series of meetings with the authors to come to a consensus on recommendations for future studies involving HRV methods in outdoor contexts.

## 3. Results

**Figure 1:**
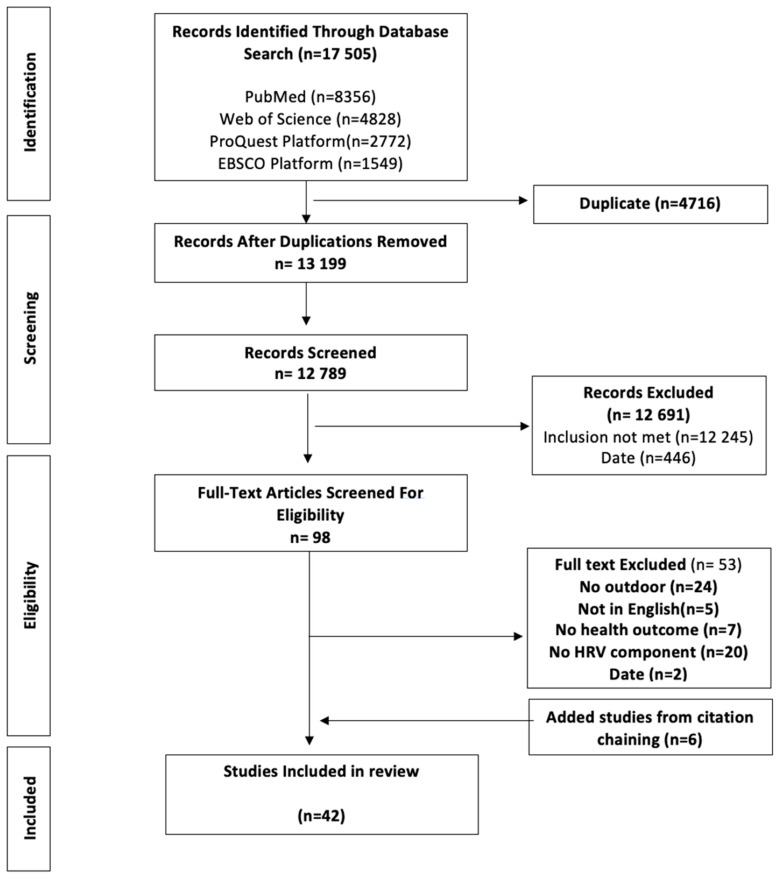
Flow Diagram and Screening Process of Included Empirical Studies.

Initial search results identified 17,505 records. Search records were imported using a reference manager (Zotero); 13,199 records remained after removing 4716 duplicates. The flow diagram of the screening process is portrayed in Figure 1. An additional 12,691 records were excluded based on a review of the title, abstract, and date. Thus, 98 articles remained for full-text review. Papers were excluded because of missing one or more of the five inclusion criteria: date range, empirical, outdoor context, HRV measures, and health outcome. The final sample of 42 papers included 34 empirical studies and eight systematic reviews. Six of the 34 empirical studies were identified after utilizing a citation-chaining process of the eight systematic reviews.

### 3.1. Descriptive Characteristics of Studies

The descriptive characteristics of HRV studies in this review are summarized in Table 2. The selected studies were published between 1996 and 2022, with 80% of these studies published in the last ten years. Most studies were published in Asian countries (Japan, Korea, and China). Twenty-seven studies focused on healthy adults, seven studies focused on adults with health issues, and eight studies were systematic reviews. A variety of study designs were used; however, the most common (n = 13) used a within-subject randomized counter-balance design. The study sample sizes were primarily small, with over half of the studies containing less than twenty participants. Twelve studies contained a sample of 21 to 50 participants, six studies contained a sample of 51 to 100, and six studies had more than 100 participants. Twenty-one studies reported the outdoor context as a rural forest environment: five were in a high altitude or mountain environment; six were in an urban forest environment; and interestingly, one was in the air involving a skydiving experience. All the empirical studies (n = 34) of the 42 included studies in the review reported ethics approval. Only 22 of the 42 studies (52%) cited the standards for HRV measurement and analysis established by the 1996 task force [11].

### 3.2. Method Characteristics

Table 3 presents the method characteristics of the HRV studies. There were seven different types of outdoor intervention; however, over half of the studies were one-day forest therapy interventions [14,16,28,29,30,31,32,33,34,35,36,37,38,39,40,41,42,43,44]. Other studies included mountaineering expeditions [41,42,43,44], forest therapy camps [45,46,47,48], green exercise [49,50,51,52], blue exercise [53], horseback riding [54], and skydiving [55]. Over half of the control groups had a similar exposure type as the intervention but in an urban context. Only nine studies relied on pre-experimental measurements. Three studies conducted their control in a lab environment, and three reported no control or comparison group.

#### 3.2.1. HRV Devices

The primary HRV device used was the Activetracer (AC301A), and twelve studies reported the use of this device [14,29,32,34,36,37,38,40,41,42,43,44,55]. One device was used in three studies, and this included the Polar Electro models, RSS800CX [49,56], and S810i [54]. The following devices were used in two studies each: HeartMath EmWave Pro [45] and PC Stress Relief System [33], Zephyr BioHarness^TM^ 3 [52,53], Actiheart [35,51], and Emotion Faros Sensor [28,49]. There were nine other devices used in only one study each, and one study did not specify the device used.

#### 3.2.2. Additional Physiological and Psychological Measures

Almost every study used other physiological or psychological measures in their study, however five studies did not report the use of any other measures [29,32,45,46,57]. The three physiological measures that were most recorded included blood pressure (Bp) [28,31,34,35,36,38,40,41,42,49,50,52,53,58], heart rate (Hr) [28,30,38,39,42,43,47,48,49,50,55,56,59,60], and salivary cortisol [33,37,39,40,41,42,44,47,49,51,54,58]. Other less common physiological measures assessed were pulse rate (PR) [31,36,41,53,58], oxygen saturation (SPo2) [56,59], and detection of natural killer cells (Nk) [48]. Self-reported construct measures varied greatly; however, the most reported measure was the Profile of Mood States (POMS), with thirteen studies using this scale [31,34,35,40,41,42,43,44,49,54,61]. Seven studies used the State-Trait Anxiety Inventory (STAI) [30,31,34,43,44,50,54], and the following self-reported scales were used in two studies each: Beck Depression Inventory (BDI) [33,47], Borgs Scale of Perceived Exertion [28,39], Digit Span Memory Test [42,47], Lake Louise Scoring System (LLSS) [57,60], Modified Semantic Differential (SD) Method [34,58], Rating of Perceived Exertion (RPE) [28,49], and Total Mood Disturbance (TMD) [39,53]. There were ten other self-report measures used in only one study each, and eight studies contained an unspecified scale or question-item survey [34,35,36,37,38,39,42,61]. Three studies did not report the use of any other self-reported measures [29,32,59].

### 3.3. HRV Analysis Methods Used

Table 4 summarizes the characteristics of HRV analysis methods used in the 34 empirical studies. Seventeen studies reported the HRV measurement period to be during the intervention [34,36,37,38,39,40,41,43,44,48,49,51,53,54,58], and 17 studies reported the measurement period as pre- post-intervention [28,31,33,35,45,46,47,49,50,52,54,55,56,57,60,62]. Interventions had a wide range of exposure times, ranging from six minutes [40] up to eighty days [45]. However, most studies (n = 20) were between 15 to 30 min for intervention exposure time [28,31,32,35,36,37,38,39,40,41,42,43,44,50,53,54,55,60]. Only six studies involved exposure time from 50 min to four hours [31,33,49,52,56], and three studies involved two to three days of exposure [46,47,48]. Two more studies involved five to 12 days [57,60], with one study reporting an 80-day exposure [45]. Twenty-five of the studies did not report the use of a relaxation period before the start of the intervention exposure [31,33,34,36,37,38,39,40,41,43,44,50,51,52,53,54,55,60]. However, three studies reported a relaxation time of five minutes [29,32,35], five studies reported a relaxation time of fifteen to thirty minutes [28,39,48,55,58], and one study reported a relaxation time of two hours (60). Recording times varied for the studies where HRV was measured during the exposure period; however, the recording times typically reflected the length of exposure. The majority of HRV recordings during exposure were between 5 and 15 min [29,32,34,37,38,40,41] or 15 and 30 min [36,39,41,51,53,56,58]. Two studies recorded for over two hours [55]. For pre- post-test designs, HRV recording times ranged from one minute [50,55] to greater than 120 min [28]. Other pre- post-intervention recording times ranged from two to five minutes [33,35,45,46,47,49,52,54,57,59,60] and 15 to 30 min [54,56], and one study did not report the length of recording time [31]. In terms of the epoch period used for HRV analysis, studies ranged from using 15 s [55] to 15 min [32], although most studies used a one-minute to four minute [34,36,37,38,40,41,45,46,50] or five-minute epoch period [33,35,47,49,52,53,54,57,59,60]. Three studies used an epoch period greater than five minutes but less than fifteen [28,49], and eight did not report their epoch period used for analysis [29,31,32,39,42].

#### HRV Analysis Software and Variables

Out of the 34 studies, 14 different types of software were used for HRV analysis. The most prominent software used was the MemCalc/Win, GMS, with thirteen studies using it [29,31,32,34,36,37,38,40,43,44,54,57]. The following software had two studies each referencing its use: Actiheart [35,55], Polar Pro Trainer [49,56] and Kubios HRV [29,39], Zephyr Technology Corporation [52,53]. Seven studies each used a different analysis software, and four studies did not report the type of analysis software used to interpret HRV data [31,34,46,50]. The most prominent HRV variable clusters used for analysis in the studies were the LF, HF, and LF/HF, with seventeen studies using this cluster set for analysis [29,31,34,36,37,38,39,40,43,44,46,55,57,58,59]. Six studies used a cluster set of LF, HF, LF/HF, SDNN, and RMSSD [38,49,53,56]; two studies used LF, HF, LF/HF, TP [35,57]; and another two used LF, HF, LF/HF, TP, SDNN, RMSSD [33,47]. Only one study each reported using the cluster sets LF, HF, LF/HF, PNN50, NN50, SDNN, RMSSD [60]; SDNN, RMSSD, SD1 [28,50]; SDNN, TP [48,53]; and RMSSD [51,55]. One study did not report the variable(s) used [45].

### 3.4. HRV Outcomes Related to ANS Activation

Only 27 of the 34 empirical studies reported health-related HRV outcomes, and Table 5 summarizes the HRV outcomes by HRV variable for these studies. Seven studies were excluded because they used HRV analysis to identify a specific physiological effect (e.g., the onset of acute mountain sickness and altitude hypoxia) rather than a health benefit/outcome. Twenty-two of the 27 studies claimed an increase in activation of the PSNS [28,29,30,33,34,35,36,37,38,40,41,43,44,47,52,53,55,58]; 18 of these studies described forest therapy as the intervention, two involved green exercise, one was described as a blue space intervention, and the final study involved skydiving. Nineteen studies claimed a decrease in the activation of the SNS [28,29,32,35,38,40,42,43,44,47,48,52,53,55,58]; 12 of these studies described forest therapy as the intervention, two involved green exercise, one was described as a blue space intervention, and the final study involved skydiving. Four studies showed no change in PSNS activation [31,33,39,49]; three described forest therapy as the intervention and one involved green exercise. Seven studies showed no change in SNS activation [31,33,37,39,49,54,58]; five described forest therapy as the intervention, one involved green exercise, and the final study involved horseback riding.

### 3.5. HRV and Altitude

Four studies used HRV methods in a high-altitude hypoxic setting [56,57,59,60], with one additional study focused on activation of the autonomic nervous system from an extreme activity, skydiving [55]. One study found that trekkers with acute mountain sickness (AMS) symptoms showed significantly lower HF results and a lower LF/HF ratio at altitude, which was reported to be a result of hypoxia [59]. A second study found that repeated exposure to altitude after experiencing AMS symptoms during a first exposure prevented any alterations in HRV when exposed the second time [57]. During a running marathon at altitude, another study found that disturbances were observed in ANS activity where SNS activity increased up to five hours post-race and vagal activity recovery after thirty hours [56]. In a study examining the different effects of altitude by sex, high altitude leads to a reduction in HRV for both sexes; however, significant differences were seen in the frequency and time domain analysis between men and women, with a significantly higher HRV found in men [60]. Authors in one study claimed that HRV is not predictive of AMS [63]. Lastly, in an extreme sport context involving skydiving, the authors claimed that there was a coactivation of the PNS and SNS [55]. The authors suggested that optimal behavioral, emotional, and cognitive functions could occur in this high-intensity setting, and this could explain the coactivation [55].

### 3.6. Study Challenges and Limitations

Table 6 summarizes the HRV-related challenges and limitations reported by the researchers of the 42 included studies: 25 studies reported no challenges, and 12 studies reported no limitations. The challenges reported in 17 studies included: psychological factors, air pollution, HRV sensor error, and exercise affecting the collection of HRV data. The limitations reported in 30 studies included: small sample sizes, age and gender not analyzed, intervention bias, fluctuations in exercise speed, insufficient information collected, environmental conditions, unconscious exposure promotion, social component confounding results, inability to replicate outdoor landscape, and no long-term follow-up measures.

### 3.7. Systematic Reviews

Eight systematic reviews were included in this review, and five focused on the health benefits of forests and outdoor environments [61,62,64,65,66,67,68]. One focused specifically on trends in Shinrin-Yoku research [64], one focused on forest therapies’ effect on hypertensive adults [69], and the last focused on forest therapy and its effect on depressive symptoms [65]. The authors from all eight reviews summarized evidence suggesting that accessing green spaces and forests is associated with health benefits. The authors of four of these reviews concluded that a relaxing effect could occur in outdoor settings, and this was supported by a decrease in SNS activity and an increase in PSNS activity [61,62,64,65]. The two reviews concerned with forest and outdoor environments [61,62] and the review related to Shinrin-Yoku [64] reported that LF, HF, and the LF/HF ratio were the most common analysis variables used. The systematic review involving hypertensive adults summarized evidence indicating that engagement in forest environments led to higher HRV for this population [69]. The authors of one review also stated that one-minute analysis intervals were good indicators of stress/relaxation phases [64]. The most common analysis software used in the studies included in this review was the MemCalc/Win, GMS, and recordings were taken before, during, and after interventions [64]. Finally, the authors of the forest therapy systematic review concluded that based on a review of the studies included in their review, the evidence was weak and there was a mixed review of positive and negative health outcomes with the use of HRV in an outdoor context.

## 4. Discussion

Our scoping review aimed to explore and compare the diverse methodological approaches used by empirical studies focusing on health-related impacts in outdoor contexts that used HRV-derived variables and analysis. This review was designed to address the scarcity of knowledge about the best practices for HRV analysis in immersive outdoor natural environments. The following three sections present a discussion related to each of the three RQs: (1) characteristics of studies using HRV methods; (2) types of HRV analysis and outcome measures; and (3) challenges and limitations reported by study authors.

### 4.1. Characteristics of Studies Using HRV Methods

Studies using HRV methods to identify the health benefits in outdoor contexts are increasing since over 80% of the studies included in this review have been published within the last ten years. This was not unanticipated, given the technological advancements resulting in the emergence of many portable/wearable devices that are available for relatively convenient and non-invasive HRV data collection [70]. In addition, most studies used additional methods to measure health (e.g., heart rate, blood pressure, psychological evaluations) along with HRV methods. Most of the interventions included within this review were related to forest bathing (Shinrin-Yoku) and were from Asian countries (Japan and Korea), where forest bathing is more commonly practiced [15]. Thus, it is likely that there are many relevant studies published in non-English languages that were not considered or included in this review. Healthy young adults were the main population of focus in the included studies, and this may simply be due to the convenience of applying new technologies to readily accessible groups of study participants. However, the effects of forest bathing or other outdoor interventions could be beneficial for older or diseased groups suffering from a variety of mental and physical illnesses [15].

In terms of study designs, there were no Randomized Control Trial (RCT) studies, and most studies followed a within-subject counter-balanced design with small sample sizes, often with less than twenty participants. The small sample size was an additional limitation, and the authors of those studies identified this in eight studies in our review. Future directions for studies investigating HRV variables in an outdoor context should seek larger sample sizes and more robust experimental designs such as Randomized Control Trials (RCTs). Additionally, future studies should explore and compare different design features such as recording before and after vs. during the intervention, optimal length of recording time, and the impact of a pre-intervention relaxation period.

The HRV devices used were varied; however, most studies (38.2%) used the Activetracer (AC301A). This device is manufactured in Japan and was primarily used in the studies related to forest bathing. Interestingly, only four studies referenced the reliability and validity of the device used and these included the Actiheart [55], Zephyr Bioharness^TM^ 3 [52], Polar Electro RSS800CX [49], and HeartMath Emwave Pro [45]. In addition to the HRV measurement device used, studies also reported a wide range of other physiological and self-report measures used (see Table 3). Study authors indicated that this helped support the HRV methods by triangulating results from these other confirmatory measures. Only two-thirds of the studies referred to the 1996 task force recommendations for HRV analysis and interpretation [11], and far less cited the actual application in their study designs. This highlights the high measurement variability and lack of consistency across studies that limits study rigor and interpretation of results. It should be noted that since the publication of the 1996 task force paper several other HRV methods papers have been published [71,72,73]. The Sassi et al. paper is a follow-up to the 1996 task force paper, specifically focusing on reviewing new analytic techniques developed since 1996 [71]. The Quintana et al. paper provides *guidelines for reporting articles on psychiatry and heart rate variability* (GRAPH), with the goal of standardizing the reporting of HRV research to develop a reliable psychiatric biomarker that improves the interpretation and reproducibility of HRV studies in psychiatry [72]. The Shaffer et al. paper provides a comprehensive summary of the metrics and norms of HRV while also providing HRV assessment strategies for clinical and optimal performance interventions [73]. The authors also cautioned that 24-h, short-term, and ultra-short-term normative values for the same derived variables are neither comparable nor interchangeable. This is particularly interesting because all three of these different measurement periods were used across the papers in our study. Finally, none of the more recent (2015–2022) papers included in our study cites any of these more recent HRV methods-related papers [71,72,73].

### 4.2. Types of HRV Analysis and Outcome Measures

The intervention exposure times varied tremendously from six minutes to 80 days; however, most studies had intervention or exposure times of 15–60 min. Surprisingly, most studies did not describe a relaxation (baseline) period before starting the HRV recording. It is known that exercise can affect HRV acutely and hours after exercise, and by not allowing for a relaxation period it is hard to determine whether the HRV results were influenced by a previous physiological or psychological state [74]. The HRV collection period for the studies was evenly distributed between recording during an intervention (n = 13) or as a pre- post-intervention design (n = 13). Recording times ranged from one minute to over two hours, with the majority falling between five and 30 min. This was aligned with the 1996 task force recommendation that short-term recordings be at least five minutes [11]. Despite multiple studies reporting a multi-day intervention, only one study had a consecutive recording time over a 24-h period.

As the 1996 task force recommended, most studies used an analysis epoch of one minute or five minutes in length for analysis [11]. However, some studies reported the use of time domain measures. Task Force recommendations suggest that time domain analysis is best with five-minute epochs taken from 24-h recordings. The most common analysis variables used in the studies were the LF, HF, and LF/HF ratio (see Table 1). This was expected given the low recording time required to gain valid results from the analysis of frequency domain variables. Additionally, most of the studies were from Japan and involved Shinrin-yoku; and the systematic review of trends in Shinrin-Yoku research also identified these three variables as most commonly used [64]. The most common software used for analysis was the Memcalc/win, GMS [64] (42% of studies). This software is also associated with the Activetracer recording device, which we know was also the most common device used (38% of studies). The HRV variables selected for analysis reflected positive changes in the ANS in most studies included in our review. Out of the 34 studies, 29 (86%) reported an increase in relaxation through activation of the PSNS, and 23 (68%) of the studies reported a decrease in stress through de-activation of the SNS.

### 4.3. Methodological Challenges and Study Limitations

The methodological challenges reported in these studies included: HRV sensor errors, exercise affecting HRV data, psychological factors, and air pollution. HRV sensor errors occurred in two studies, and in these studies the leads connecting the portable HRV device became disconnected. Exercise affects cardiovascular activity, and an increase in physical activity increases heart rate. This increase in heart rate from activities as simple as walking in a forest environment also impacts HRV [71]. Given that most studies reported an increase in activity or exercise during their interventions, it is difficult to determine if changes in HRV came from the outdoor environment, or from other confounding factors such as psychological state and exercise. Thus, more research is needed to analyze and understand HRV-related outcomes in states other than resting, where the heart rate is elevated due to physical exertion.

One psychological factor that could influence HRV data is related to the concepts or psychological states of biophilia and biophobia. *Biophilia* is a theory that suggests humans have an inherent curiosity to seek connections with nature and other forms of life, and contrarily, *biophobia* is simply a fear of nature resulting in an aversion from it [75]. Several studies in our review referred to biophilia and/or biophobia, and it could be that psychological states such as these could have an impact on HRV-related outcome data. For instance, it could be that a person exhibiting biophilia feelings may feel more connected and attracted to nature, and this may lead to activation of the PSNS. Conversely, a person exhibiting biophobia feelings may demonstrate higher stress levels while in nature, and this may activate the SNS. There was no psychological test implemented in any of the studies to assess the presence of either of these states, and it is therefore unknown if they influenced study results. Interestingly, air quality was only reported in three studies even though it can have a substantive influence on HRV data [37,64,76]. Air composition and quality can have various effects on the pulmonary and cardiovascular systems, and this may impact results from studies based in a forest setting compared to an urban setting where air quality is much lower [77].

There were 10 distinct limitations identified by the authors of most of the included studies; however, no limitations were identified in 12 of the studies. Many studies in our review contained small sample sizes, and this precluded sub-analysis by age and gender [78]. The samples for most of the studies were healthy university students, which suggests the need to assess other populations [79]. Additionally, some studies just contained one specific sex. It is known that HRV does vary between males and females, such as men having a higher HRV on average [78]. Unconscious promotion of the exposure or intervention may have occurred in two studies where investigators were required to guide participants along a trail [29,49]. Inability to replicate the outdoor landscape was a limitation identified by authors in one study where a treadmill was used for one of the control groups where the intervention was mountain hiking [49]. The treadmill did not have a decline function and it did not reflect the many obstacles and diverse uneven terrain associated with mountain hiking [49]. Another limitation was selection bias since study participants recruited for some studies may have self-selected to participate because they appreciated outdoor environments and or expected to have feelings of biophilia. Fluctuations in speed was another limitation because it is very hard to control in many outdoor environments [28,65], whereas in more controlled urban settings (comparison groups), the speed was easy to set given the relatively uniform terrain. In one study, there was a substantive social component connected with the intervention, and this could have potentially increased the enjoyment or lack of enjoyment of the experience [47]. Environmental conditions were also presented as a limitation, although all studies were conducted in relatively good weather [80]. Different seasons, environmental conditions and temperatures could all have had a result on the effect of the outdoor exposure [35]. Varying environmental factors can also affect a person’s psychological enjoyment of the outdoors [80]. Finally, none of the studies used long-term follow-up measures, and this precluded any investigation into the longevity of any measured HRV-derived effects.

## 5. Conclusions

Our scoping review identified a wide variety of methodological approaches using HRV collection and analysis in outdoor contexts. The findings summarized the characteristics of HRV studies used in outdoor contexts, the methods used for HRV collection and analysis, and the limitations and challenges identified by the authors in the included studies. From our scoping review, five recommendations were created by our research team for consideration in future research study designs using HRV methods in outdoor and health-related contexts:

(1) Researchers should follow the standards of HRV analysis outlined in the 1996 task force paper and should also refer to more recent standards such as those provided by Sassi et al., 2015, Quintana et al., 2016, and Shaffer et al., 2017. To our knowledge, these are the most current standards on HRV collection, analysis, and interpretation. To increase the validity and replicability of future results, new studies should adhere to these guidelines.
(2) It is also recommended that studies increase their methodological rigor by increasing sample size and implementing stronger study designs such as randomized control trials with more varied populations (other than university students).
(3) Validity and reliability confirmation need to be completed and reported on the devices being used for HRV recordings as well as the analysis software interpreting the collected data. There were many devices and analysis software used, and very few studies reporting or referencing validation of their use.
(4) Many studies identified the biophilia/biophobia theory yet had no psychological evaluation or assessment in place to account for it. More research should be devoted to investigating this theory and the possible impacts it can have on results, considering its potential influence on stress and relaxation states. It should also be noted that there were a wide range of self-report psychological and confirmatory scales used, and the psychometric properties of these scales should be reported.
(5) Finally, there is a limited understanding of the effect of exercise on HRV while in free-living conditions such as being in nature. Given the outdoor context and intervention (walking) in most of these studies, a greater understanding is required to identify the interaction effect between the outdoor environment and varying levels of physical activity and how they interact to impact HRV.

HRV methods do hold promise in the future for determining health-related outcomes in outdoor free-living contexts, but caution is warranted in reviewing current study claims.

## Figures and Tables

**Table 1:** Main HRV Analysis Variables.

HRV Variable	Short Form	Common Use
Time Domain		
Standard Deviation of N-N Intervals	SDNN	Examining both long- and short-term variability, reflecting overall HRV
Root Mean Square of Successive Differences in N-N Intervals	RMSSD	Examining short-term effects directly reflecting activation of the PSNS
Proportion of Differences in Consecutive N-N Intervals that are Longer than 50 ms	PNN50	Examining activation of the PSNS
Number of Adjacent N-N that Differ from each other by more than 50 ms	NN50	Examining activation of the PSNS
Poincare Plot Standard Deviation Perpendicular to the Line of Identity	SD1	Examining short-term HRV
Frequency Domain		
High Frequency (0.15–0.40 hz)	HF	Examining activation of the PSNS
Low frequency (0.04–0.15 hz)	LF	Examining activation of both PSNS and SNS
Low Frequency/High Frequency Ratio	LF/HF	Determining the predominant activation of PSNS or SNS
Total Power	TP	Examining overall autonomic activity

**Table 2:** Descriptive Characteristics of HRV Studies in Outdoor Contexts.

Characteristics	N (%)
Date of Publication (n = 42) *	
2020–2022	10 (23.8)
2010–2019	26 (61.9)
2000–2009	6 (13.3)
1996–1999	0 (0.0)
Type of Participant (n = 34) *	
Healthy Adults	27 (79.4)
Adults with Health Issues	7 (20.6)
Country (n = 42) *	
Japan	17 (40.5)
Korea	5 (11.9)
United Kingdom	2 (4.8)
Taiwan	1 (2.4)
Austria	1 (2.4)
Denmark	3 (7.1)
Germany	1 (2.4)
Czech Republic	1 (2.4)
Ukraine	1 (2.4)
Finland	2(4.8)
Spain	2 (4.8)
USA	3 (7.1)
China	2
Study Design (n = 34) *	
Within-Subject Randomized Counter-Balanced	13 (38.2)
Randomized Cross-Over	8 (23.5)
One Group Pre- Post-Test	9 (26.5)
Non-Equivalent Control Group	3 (8.8)
Experimental design	2 (5.9)
Sample Size (n = 34) *	
≤20	10 (29.4)
21–50	12 (35.3)
51–100	6 (17.6)
>100	6 (17.6)
Outdoor Context (n = 34) *	
Rural Forest	21 (61.8)
Mountain	5 (14.7)
Urban Forest	6 (17.6
Blue Space	1 (2.9)
Air (Sky Diving)	1 (2.9)
Ethics Reported in Study (n = 42) *	
Yes	34 (81.0)
No	8 (19.0)
Cited Task Force Standards (n = 42) *	
Yes	22 (52.4)
No	20 (47.6)

* n = 42 includes 8 systematic reviews and n = 34 includes empirical studies only.

**Table 3:** Method Characteristics of HRV Studies in Outdoor Contexts.

Method Description	N (%)
Intervention Description (n = 42) *	
One-Day Forest Therapy	19 (45.2)
Mountaineering Expedition/Altitude Sport	4 (9.5)
Forest Therapy Camp	4 (9.5)
Green Exercise	4 (9.5)
Blue Space	1 (2.4)
Horseback Riding	1 (2.4)
Skydiving	1 (2.4)
No Intervention (Systematic Review)	8 (19.0)
Control or Comparison (n = 34) *	
Urban Setting	19 (55.8)
Pre-Experimental Measurements	9 (26.5)
Laboratory Setting	3 (8.8)
No Control	3 (8.8)
HRV Device Used (n = 34) *	
Activetracer AC-301A	13 (38.2)
Polar Electro RSS800CX or S810i	3 (8.8)
HeartMath EmWave Pro and PC Stress Relief System	2 (5.9)
Zephyr BioHarness™ 3	2 (5.9)
Actiheart	2 (5.9)
eMotion Faros Sensor	2 (5.9)
HRV4training	1 (2.9)
BodyGuard2 device–First Beat	1 (2.9)
uBioMacpa(Biosense creative, Seoul, Korea)	1 (2.9)
CheckMyHeart	1 (2.9)
Custo Cardio 100/110 BT	1 (2.9)
Gem Heart Cardiolight	1 (2.9)
LR-8Z11	1 (2.9)
myBeat Electrocardiogram	1 (2.9)
T-REX TRI00A	1 (2.9)
Unspecified	1 (2.9)
Additional Physiological Measures Used (n = 34) *	
Blood Pressure	15 (44.1)
Heart Rate	14 (41.2)
Salivary Cortisol	13 (38.2)
Pulse Rate	5 (14.7)
Oxygen Saturation	2 (5.9)
Natural Killer Cells	1 (2.9)
No other Physiological Measures Used	5 (14.7)
Additional Self-Reported Measures Used (n = 34) *	
Profile of Mood State (POMS)	13 (38.2)
State-Trait Anxiety Inventory (STAI)	7 (15.4)
Beck Depression Inventory (BDI)	2 (5.9)
Borgs Scale of Perceived Exertion	2 (5.9)
Digit Span Memory Test	2 (5.9)
Lake Louise Scoring System (LLSS)	2 (5.9)
Modified Semantic Differential (SD) Method	2 (5.9)
Rating of Perceived Exertion (RPE)	2 (5.9)
Total Mood Disturbance (TMD)	2 (5.9)
SF36 Health Survey	2 (5.9)
Well-Being Manifestation Measure Scale	1 (2.9)
EuroQol Visual Analog Scale (EQ-VAS)	1 (2.9)
Hamilton Rating Scales for Depression (HRSD)	1 (2.9)
Y-OQ-SR 2.0	1 (2.9)
Y-OQ 2.01	1 (2.9)
Maslach Burnout Inventory-General Survey (MBI-GS)	1 (2.9)
Montgomery–Asberg Depression Rating Scales (MADRS)	1 (2.9)
Perceived Restorative Scale (PRS)	1 (2.9)
Stress Response Inventory (STI)	1 (2.9)
Sensation Seeking Scale-V questionnaires	1 (2.9)
Visual Analogue Scale (Unspecified)	2 (598)
Unspecified Scales and Questions	8 (23.5)
No Self-Reported Measures Used	3 (8.8)

* n = 42 includes 8 systematic reviews and n = 34 includes empirical studies only.

**Table 4:** Characteristics of HRV Analysis Methods and Outcome Variables.

Analysis and Outcome Variable Description	N (%)
HRV Data Collection Period (n = 34)	
During Intervention/Control Period	17 (50.0)
Before and After Intervention/Control Period	17 (50.0)
Intervention Exposure (n = 34)	
6 min	1 (2.9)
15–30 min	20 (58.8)
60–240 min	6 (17.6)
2–3 days	3 (8.8)
5–12 days	3 (8.8)
80-day	1
Relaxation Time before Measurement (n = 34)	
5 min	3 (8.8)
15–30 min	5 (14.7)
120 min	1 (2.9)
Not Reported	25 (73.5)
Total Recording Time (n = 34)	
1 min	2 (5.9)
3 min	2 (5.9)
5 min	10 (29.4)
15 min	9 (26.5)
>15–30 min	8 (23.5)
>120	2 (5.9)
Not Reported	1 (2.9)
Epoch Used for Analysis (n = 34)	
15 s	1 (2.9)
1 min	7 (20.6)
3 min	3 (8.8)
5 min	10 (29.4)
>5–15 min	3 (8.8)
Not Reported	10 (23.5)
Analysis Software Used (n = 34)	
MemCalc/Win, GMS	13 (38.2)
Actiheart	2 (5.9)
Polar Pro Trainer	2 (5.9)
Kubios HRV	2 (5.9)
EmWave PC Stress Relief System	2 (5.9)
Zephyr Technology Corporation	2 (5.9)
(RRI) T-REX^®^ (Monitor and Care)	1 (2.9)
First Beat Technologies	1 (2.9)
ElectroCardiograph (Cardio CE Bt)	1 (2.9)
HeartMath LLC	1 (2.9)
CheckMyHeart Plusi R30 V4	1 (2.9)
Gem Heart Cardiolight	1 (2.9)
KyPlot 5.0, Kyence Lab Inc.	1 (2.9)
Not Reported	4 (5.9)
HRV Analysis Variables Used (n = 34)	
Time Domain n = 12 (%)	
RMSSD	10(83.3)
SDNN	9 (75.0)
SD1	2 (16.7)
NN50	2 (16.7)
PNN50	1 (8.3)
Frequency Domain n = 29 (%)	
LF	25 (86.2)
HF	25 (86.2)
LF/HF	25 (86.2)
TP	6 (20.7)
Not Reported	1 (2.9)
HRV Analysis Clusters (n = 34)	
LF, HF, LF/HF	18 (52.9)
LF, HF, LF/HF, SDNN, RMSSD	4 (11.8)
LF, HF, LF/HF, TP	2 (5.9)
LF, HF, LF/HF, TP, SDNN, RMSSD	2 (5.9)
LF, HF, LF/HF, PNN50, NN50, SDNN,	1 (2.9)
RMSSD	2 (5.9)
SDNN, RMSSD, SD1	2 (5.9)
SDNN, TP	2 (5.9)
Not Reported	1 (2.9)

**Table 5:** Summary of HRV Outcomes by HRV Variable and ANS Activation (N = 27).

	Frequency Domain				Time Domain		
	LF N = 22 (%)	HF N = 22 (%)	LF/HF N = 22 (%)	TP N = 6 (%)	SDNN N = 8 (%)	RMSSD N = 8 (%)	SD1 N = 1 (%)
Increase	1 (4.5)	16 (72.7)	0 (0.0)	5 (83.3)	7 (87.5)	6 (75.0)	1 (100.0)
Decrease	15 (68.2)	3 (13.6)	16 (72.7)	0 (0.0)	0 (0.0)	0 (0.0)	0 (0.0)
No Change	6 (27.2)	3 (13.6)	6 (27.2)	1 (16.6)	1 (12.5)	2 (25.0)	0 (0.0)
	**Activation of PNS (26)**				**Activation of SNS (26)**		
Increase	22 (84.6)				0 (0.0)		
Decrease	0 (0.0)				19 (73.1)		
No Change	4 (15.4)				7 (26.9)		

**Table 6:** Challenges and Limitations of HRV Studies in Outdoor Contexts.

Challenges (N = 39)	N (%)
Psychological Factors	7 (16.7)
Air Pollution	3 (7.1)
HRV Sensor Error	2 (4.8)
Exercise Affecting HRV Data	2 (4.8)
None Reported	25 (59.5)
**Limitations (N = 55)**	**N (%)**
Small Sample Size	10 (23.8)
Age and Gender Not Analyzed	9 (21.4)
Selection Bias	3 (7.1)
Fluctuations in Exercise Speed	3 (7.1)
Insufficient Information Collected	3 (7.1)
Environmental Conditions	4 (9.5)
Unconscious Promotion of Exposure	2 (4.8)
Social Component Confounding Results	2 (2.4)
Inability to Replicate Outdoor Landscape	2 (2.4)
No Long-Term Follow-Up Measures	2 (2.4)
None Reported	12 (28.8)

Note: Counts in this table refer to specific challenges and limitations cited in the studies, and several could be cited in a particular study.

## Data Availability

References are provided for all studies included in this review.

## Appendix A

**Table A1:** HRV Search String.

Concept Number	Concept Name	Search String
**1**	**HRV**	(HRV or “Heart rate variability” or “HR variability” not “human rhinovirus”) OR (“Heart Rate”[Mesh] and variability) OR (“R-R interval” OR “R-R Variability” OR “RR interval” OR “RR Variability”)
**2**	**Outdoors**	(“nature”[MeSH Terms] or “wilderness”[MeSH Terms] or “environment”[MeSH Terms]) or (“natural setting*” or “natural environment*” or “in nature”) or (“wild*”) Or (“outdoor*” or “out-door” or “out door” or “out-of-doors” or “out of doors”) Or (“outside”) Or (“green” or “greenspace” or “greenery”) Or (“flora” or “fauna” or “countryside” or “landscape” or “open air” or “hik*” or “camp*” or “backwoods” or “hill*” or “mountain*” or “forest*” or “wood*” or “garden*” or “park*”)
**3**	**HRV and Outdoors**	(((((((((“nature”[MeSH Terms] or “wilderness”[MeSH Terms] or “environment”[MeSH Terms])))) OR (“wild*”)) OR (((“outdoor*” or “out-door” or “out door” or “out-of-doors” or “out of doors”)))) OR (“outside”)) OR (((“green” or “greenspace” or “greenery”)))) OR (((“flora” or “fauna” or “countryside” or “landscape” or “open air” or “hik*” or “camp*” or “backwoods” or “hill*” or “mountain*” or “forest*” or “wood*” or “garden*” or “park*”)))) OR natur*) AND (((HRV or “Heart rate variability” or “HR variability” not “human rhinovirus”) OR (“Heart Rate”[Mesh] and variability) OR (“R-R interval” OR “R-R Variability” OR “RR interval” OR “RR Variability”)))
